# *Vicia faba* peel extracts bearing fatty acids moieties as a cost-effective and green corrosion inhibitor for mild steel in marine water: computational and electrochemical studies

**DOI:** 10.1038/s41598-022-24793-3

**Published:** 2022-11-29

**Authors:** Khaled A. Abdelshafeek, Walid E. Abdallah, Wael M. Elsayed, Hassan A. Eladawy, A. M. El-Shamy

**Affiliations:** 1 Pharmaceutical Industries Division, Chemistry of Medicinal Plants Dept., National Research Center, El-Bohouth St. 33, Dokki, Giza, 12622 Egypt; 2 Chemistry Department, Faculty of Science, Al-Azhar University, Cairo, Egypt; 3 Physical Chemistry Department, Electrochemistry and Corrosion Lab., National Research Centre, El-Bohouth St. 33, Dokki, Giza, 12622 Egypt

**Keywords:** Chemistry, Engineering, Materials science

## Abstract

The goal of this research is to determine what chemicals are present in two different extracts (hexane and acetone) of Vicia faba (family Fabaceae, VF) peels and evaluate their effectiveness as a corrosion inhibitor on mild steel in a saline media containing 3.5% sodium chloride. Gas chromatography-mass spectrometry (GC/MS) was used to determine the composition of various extracts. It was determined that fourteen different chemicals were present in the hexane extract, the most prominent of which were octacosane, tetrasodium tetracontane, palmitic acid, and ethyl palmitate. Heptacosane, lauric acid, myristic acid, ethyl palmitate, and methyl stearate were some of the 13 chemicals found in the acetone extract. Using open circuit potential, potentiodynamic polarisation, and electrochemical impedance spectroscopic techniques, we can approximate the inhibitory effects of (VF) extracts on mild steel. The most effective inhibitory concentrations were found to be 200 ppm for both the hexane and acetone extracts (97.84% for the hexane extract and 88.67% for the acetone extract). Evaluation experiments were conducted at 298 K, with a 3.5% (wt/v) NaCl content and a flow velocity of about 250 rpm. Langmuir adsorption isotherm shows that the two extracts function as a mixed-type inhibitor in nature. Docking models were used to investigate the putative mechanism of corrosion inhibition, and GC/MS was used to identify the major and secondary components of the two extracts. Surface roughness values were calculated after analyzing the morphology of the metal's surface with and without (VF) using a scanning electron microscope (SEM). The results showed that throughout the surface of the mild steel, a thick adsorbate layer was formed. Quantum chemical calculations conducted on the two extracts as part of the theoretical research of quantum chemical calculation demonstrated a connection between the experimental analysis results and the theoretical study of the major chemical components.

## Introduction

Even though agricultural and organic wastes, such as bean husks (*Vicia faba* peel), are categorized as environmental pollutants, these materials may be put to use to solve a variety of issues, such as preventing corrosion in several different kinds of steel. Because plants are rich sources of bioactive metabolites, traditional systems of folk medicine have relied heavily on a variety of plants for the treatment of a wide range of conditions ever since ancient times^1^. The secondary metabolites of plants and animals are created by metabolic pathways and are present as small components of plant tissues. The types of secondary metabolites and the amounts of those secondary metabolites in various regions of the plant vary greatly^2^. The *Vicia faba* bean, often known as the *faba* bean, is a member of the legume family (Fabaceae) and is considered a vital food and fodder legume crop across the globe^3^. In the year 2010, it was stated that the Faba bean vegetable, which is cultivated primarily as a spring crop in China, India, South America, Middle-Eastern Europe, and the Mediterranean region, is considered to be the primary source of nutrition for plants, particularly within the River population^4,5^. The waste of fruit and vegetable peels, which may be found in both commercial and domestic kitchens, is responsible for a significant amount of nutrient and economic loss as well as environmental difficulties^6^. The indigestible parts of fruit and vegetables that are thrown away contain valuable high amounts of phytochemical constituents and essential nutrients such as carotenoids, enzymes, polyphenols, oils, vitamins, and a wide variety of other compounds. These compounds are present in peels, seeds, and other constituents of vegetables and fruits that are commonly used^7^. Identification of seventeen compounds (85.97%) by GC/MS analysis of hexane extract of *faba* bean peels, as well as identification of seventeen phenolics and sixteen flavonoids by the HPLC analysis of the ethyl acetate fraction of the *V. faba* peels. GC/MS analysis of hexane extract of *faba* bean peels. Additionally, two flavonoid substances were extracted from the skin of the fruit^8^. Utilization of these wastes has been shown in numerous studies as a means of reducing environmental pollution and deriving advantages from the components that constitute their waste: they include medical and industrial benefits, in addition to investigations into their chemical make-up. The bioactive compounds of fruit peels are an important applicable source in a variety of industries, including the food industry for the development of edible films, the food industry for the production of probiotics, the cosmetics industry, the textile industry, the pharmaceutical industry, and various other industries for the production of valuable products. The low-cost horticulture wastes might be employed as a unique stage in the process of generating the value-added product as part of its sustainable usage. Corrosion of metals is a significant issue that affects many different businesses and is estimated to cost billions of dollars each year. This issue also has economic repercussions. The use of certain additives that are safe for human health to minimize the corrosive attack of solutions to the contacted metallic materials is one of the most efficient strategies to avoid metal corrosion and reduce the corrosive attack of solutions to the contacted metallic materials^9^. More attention has been paid to the search for environmentally friendly corrosion inhibitors and to the study of plant extracts as a means of resolving issues that have been brought about as a result of the high cost, high toxicity, and high environmental risk posed by synthetic chemical corrosion inhibitors. Because of their cheap cost, biodegradability, high availability, and non-toxic nature, inexpensive and ecologically friendly plant extracts might be employed as corrosion inhibitors^10^. In addition, these plant extracts are environmentally beneficial. The purpose of this study was to investigate the phytochemical constituents of various extracts of *Vicia faba* peel, as well as their inhibiting effect on corrosion using electrochemical techniques, surface characterization using SEM&EDS, and assessment of their biocidal activity. Additionally, the study aimed to evaluate the phytochemical constituents of different extracts of *Vicia faba* peel.

## Corrosion overview

Problems of essential importance, resulting in large economic losses on a global scale, are brought on by corrosion^11–15^. Pure metals undergo an electrochemical reaction when exposed to a salty media, which results in the formation of chemically more stable compounds such as oxide, hydroxide, or sulfide^16–19^. This makes the process thermodynamically plausible and since it is useful in a wide variety of technical applications, mild steel is used in many different industries, including the automotive, aerospace, petroleum production and refining, maritime application, chemical, and military sectors^20^. In a solution containing 3.5% NaCl, the metal loss is rather considerable in a short amount of time, and we can gravimetrically assess very fast whether or not an inhibitor effect is there^21^. It is crucial to examine structural materials that are exposed to outside conditions, particularly in saline media, where corrosion is substantially more severe than in other environments. Mild steel is put to widespread use in industrial settings due to the ease with which it can be obtained, as well as its malleability, ductility, and high tensile strength. Because of their low cost, ease of use, and high level of efficacy, the use of inhibitors is one of the most effective and economical strategies to regulate the dissolution of metals^22^. Several different organic compounds, such as amines and their derivatives, macrocyclic compounds, open-chain aromatic Schiff bases, Mannich bases, and others, have been reported as potential inhibitors of corrosion; however, it is common knowledge that these compounds are extremely harmful to the environment^23^. Other organic compounds that have been reported as potential inhibitors of corrosion include several toxic compounds which have a bad effect on the environment^24^. The production of corrosion inhibitors that are less harmful to the environment remained a significant obstacle for current research directions. Several natural compounds have been evaluated as potential green corrosion inhibitors for mild steel in acidic and salty environments. Some of these natural products include tobacco, black pepper, lignin, and castor oil seeds. The natural product extracts were widespread around the globe, and it is safe and non-toxic for usage in saltwater and/or freshwater as a corrosion inhibitor for mild steel^25^. Additionally, natural product extracts were widely available. The research was conducted on the effectiveness of water and solvent extracts of natural products as corrosion inhibitors for mild steel pipes that were utilized and used in various corrosive conditions^26^. The temperature dependence of natural product extracts as a corrosion inhibitor as well as theoretical calculations that were supported by taking into consideration major chemical components as active corrosion inhibitors for comparison and correlations with the experimental data was considered to be the most important points of this research article. In this respect, the inhibitory activity of hexane and acetone extracts of (VF) function as anti-corrosion materials for mild steel specimens in 3.5% NaCl. These results were determined after the specimens were immersed in the test solution^27^. The classic electrochemical methods of OCP, PD, and EIS were used to conduct corrosion experiments, and the kinetic and thermodynamic process parameters were investigated at a range of temperatures. In addition, the findings from the experiment are associated with the analysis of the theoretical calculation, which provides compelling proof of the inhibitive effect^28^. Vicia faba (VF) extract is the name of the herbal product that was used in the formulation of the (VF) extracts, and the purpose of this work is to evaluate the inhibition performance of the (VF) extracts on the corrosion of mild steel in a 3.5% NaCl solution. This evaluation is based on an environmentally friendly approach. Fatty acids are the phytochemical that represents the most often found components of (VF). In comparison to several other published papers that have a natural origin, the chemical structure of the components of (VF) achieves a high level of success in meeting the requirements of corrosion inhibitors. In addition to this, the plant-based source of (VF) is regarded as a cost-effective source for corrosion inhibition methods, and the phytochemicals it contains are of moderate to complex molecular size and include a large number of potential active adsorption sites. Importantly, in this paper, we investigate the possibility of a dual-function work that would involve both the secure disposal of a cost-effective herbal source and its use in industrial applications as an environmentally friendly corrosion inhibitor. Both of these functions would be performed simultaneously. So, this study aims to identify the chemical constituents of different extracts of *Vicia faba* peels and evaluation of their activity as corrosion inhibitors on mild steel in a saline medium containing 3.5% sodium chloride^29^.

## Material and methods

### Materials

#### Plant material

In January 2021, the fresh fruit of the *Vicia faba* plant was procured from the many marketplaces located within the governorate of Sharkia in Egypt. It was then peeled, and the peels were let dry in the shade for ten days, switching positions each day. After the peels were fully dried, they were finely ground up for the extraction process and stored in bags that were sealed^30^.

#### Extracts, electrolytes, and inhibitors preparation

The dried powdered peels of (VF) (500 g) were individually defatted with n-hexane in a Soxhlet for two days until the solvent was exhausted. This produced an extract with a greenish tint. A part of this extract was filtered through fuller's earth to remove the colored pigments and produce an extract that was transparent and yellowish in hue. After the powdered peels had been defatted, they were extracted with acetone multiple times (4 × 400 ml). The solvents were evaporated at 35 °C under reduced pressure to afford the hexane and acetone extracts (5 g and 6 g respectively) which were analyzed using GC/MS^31^. Table 2 provides the GC/MS data of chemical structures of some of the most prevalent classified functional categories of (VF) extract active components. After diluting the extract with 3.5% NaCl, we were able to create test solutions with varying concentrations of (VF) extract. These concentrations were 50, 100, 150, and 200 ppm, respectively. The corrosive solution, which had 3.5% sodium chloride in it, was made by mixing laboratory grade, 98% sodium chloride from Sigma-Aldrich with distilled water. Different amounts of the inhibitor were added to the solutions of 3.5% sodium chloride that were made (50–200 ppm).

#### Analysis of metallic electrode

The nominal composition of the mild steel rod with a diameter of 1 cm that was employed in this investigation may be found in Table 1. To conduct electrochemical research, the mild steel was incised and sectioned using a Clarke power hacksaw and an ESM 700 excel shaping machine. This produced specimens of mild steel with an average length of one centimeter that was used as test specimens. Following the application of solder to cover copper wires used in electrical connections, the identical steel specimens were mounted over epoxy resin with a lay-open zone of 1 cm^2^. Emery paper of grades 100, 320, 600, 800, 1000, and 1200 was used to mechanically abrade the surface of the working electrode before any measurements were taken. This was done before any measurements were taken. Following that, the specimens made of mild steel were cleaned with distilled water and acetone, and then they were dried using warm airflow.

**Table 1:** elemental analysis results of mild steel sample by XRF.

Metal	Fe	Si	Mn	P	S	Al	C
Wt%	99.438	0.08	0.36	0.02	0.018	0.004	0.08

After being cleaned and polished using emery paper numbered 400, 600, 800, and 1000, the electrodes were then degreased with acetone and allowed to air dry.

### Methods

#### GC/MS analysis

The following are the parameters for the gas chromatography–mass spectrometry instrument that was used for the GC–MS analysis of volatile compounds in n-hexane and acetone extracts. This instrument was located in the Department of Medicinal and Aromatic Plants Research at the National Research Center in Dokki, Giza, Egypt. The instrument was a TRACE GC Ultra Gas Chromatograph, which was manufactured by THERMO Scientific Corp. in the United States. It was linked with a THERMO mass spectrometer detector (ISQ Single Quadrupole Mass Spectrometer). The GC–MS system has been built with a TG-5MS column that is 30 m long and has an internal diameter of 0.25 mm. The film thickness is 0.25 µm. The research was carried out using helium as the carrier gas at a flow rate of 1.0 ml/min and a split ratio of 1:10. The following temperature program was used throughout the process. At 60 °C for 1 min, then gradually increasing to 240 °C at a rate of 3.0 °C per min while maintaining that temperature for 1 min. Both the injector and the detector were maintained at 240 °C. In all, 0.2 µl worth of samples diluted in hexane at a ratio of 1:10 were injected. Electron ionization (EI) was used at 70 eV to get the mass spectra, and the spectral range used was 40–450 m/z. The majority of the compounds were identified by comparing their retention indices relative to C7–C44 (the n-alkane series), as well as by comparing their mass spectra fragmentation and retention times with those that were already reported in the Wiley spectral library collection and the NSIT library, in addition to the existing body of literature^32^. By comparing the mass spectra of the various components, the individual components have been identified^33^.

#### Electrochemical study

To carry out the electrochemical studies, a conventional 3-electrode cell assembly was used. This cell assembly is comprised of mild steel with a surface area of 1 cm^2^ that acts as a working electrode, Ag/AgCl (silver/silver chloride reference electrode), also known as the reference electrode, which is coupled to the cell on the exterior through lugging capillary, the tip of which was placed near the working electrode, and platinum mesh, which is the counter electrode^30^. The electrochemical study was carried out at room temperature using a corrosive medium consisting of a 100 ml solution of 3.5% sodium chloride that was maintained in a stirred state at a rate of 250 revolutions per min. To generate stable OCP, the cell assembly was agitated for 60 min before the polarization data was collected (open circuit potential). An estimate of EIS was also performed at 298 K at the frequency in the range of (100 kHz–10 MHz) with a magnitude of 4 mV peak to peak using AC signals at stable OCP. This estimation was carried out with the oscilloscope calibrated to OCP. CHI Instruments Electrochemical Workstation was used for carrying out electrochemical experiments, which included sweeping the potential between − 200 and + 200 mV from OCP at a scan rate of 0.001 Vs^−1^ (Model 660C). The Tafel extrapolation curve was used to get the values for corrosion potential (E_corr_) and corrosion current density (i_corr_)^34^. The inhibition efficiency was computed using the following Eq. (1)

1 $$\upeta \left(\mathrm{\%}\right)=\frac{{\mathrm{i}}_{\mathrm{corr}}^{0}-{\mathrm{i}}_{\mathrm{corr}}}{{\mathrm{i}}_{\mathrm{corr}}^{0}}\times 100$$

where i^0^_corr_ & i_corr_ represent corrosion current densities without and with inhibitors, respectively.

#### Surface characterization

After exposing the metal specimens to a medium containing 3.5% NaCl for 24 h, immersing them in blank circumstances, and having the presence of 200 ppm extract for each solvent, a scanning electron microscopy study was carried out to investigate the shape of the corroded surface. The microstructural pictures were acquired for samples of mild steel that had been submerged in a solution with or without 3.5% NaCl + 200 ppm extracts of hexane and/or acetone^35^.

#### Computational study

Density functional theory (DFT) with unrestricted spin using DMol_3_ as an implemented module in Materials Studio software (Accelrys Inc.) was applied. “GGA” Generalized gradient approximation of the “B3LYP” Becke3–Lee–Yang–parr level using 6-311G** basis set. The chemical reactivity parameters have been computed for AO and HERA, as; “S; softness (measurement of molecules' stability), η; hardness (reverse of softness), µ; molecules' chemical potential, χ; electronegativity (grabbing-electrons-power), μ− & μ+; electronic affinity transfer & accept respectively, ω− & ω+; molecule suitability for providing & gain an electron respectively, ωi; electrophilicity index (evaluating the relative strengths of electron donors and acceptors), ΔN_max_ = χ/2η (highest amount of electrons that can be exchanged in a chemical reaction), I; ionization-potential, A; electron affinity^36^.

#### Molecular dynamic (MD) simulations

MD can be simulated an adsorption behavior of Fatty acid inhibitor into a mild steel surface using the Adsorption-Locator-module (Materials studio). Fe (1 0 0) plane with simulation box (22.90 Å × 57.26 Å × 26.68 Å) was applied in the adsorption process simulation. “E_adsorption_” interaction energy between Fe and Fatty acid inhibitors was equatorially represented “E_adsorption_ = E_total_ − (E_Fe_ + E_inhibitor_)”, while “E_total_” total energy of crystal combined with inhibitor and E_inh;_ inhibitor energy, E _Fe;_ iron surface energy. The COMPASS force field was used to optimize the adsorption system^37^.

### Legislation of experimental research

The collection of plant material complies with relevant institutional, national, and international guidelines and legislation. All authors confirm that all methods were carried out following relevant guidelines in the “Methods” section.

## Results and discussion

### Identification of chemical constituents of different extracts

**Figure 1:**
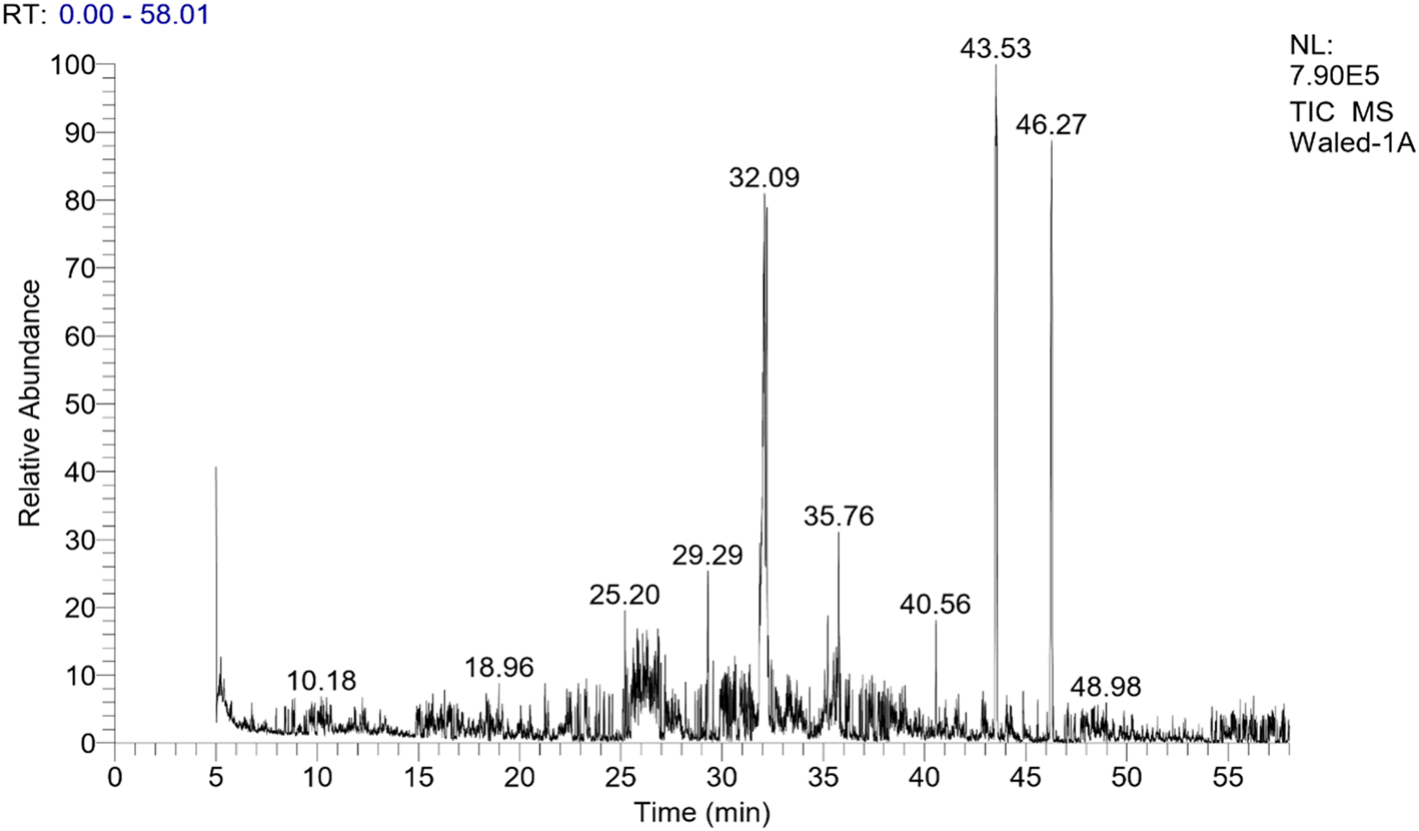
GC chromatogram of hexane extract of Vicia faba peel.

**Figure 2:**
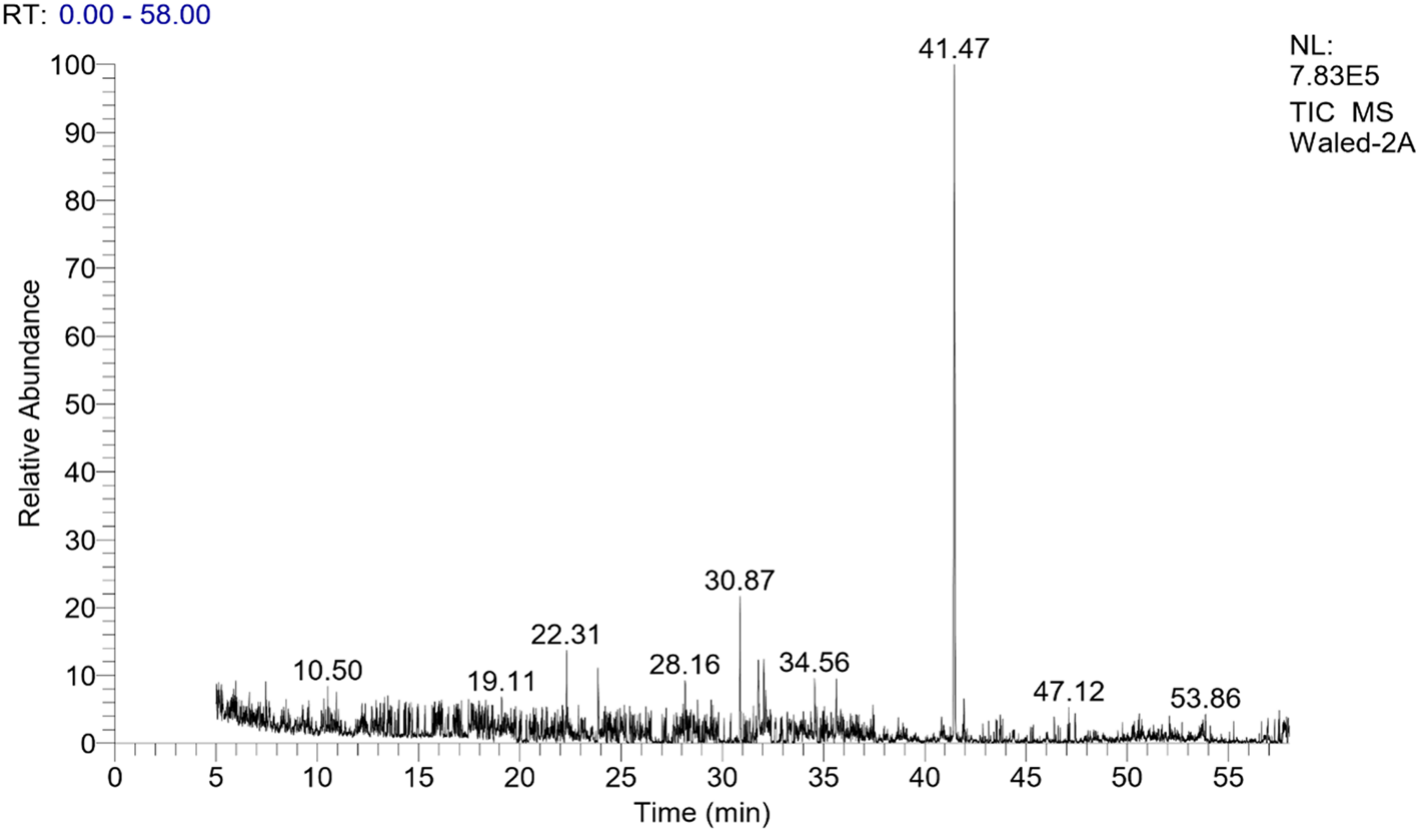
GC chromatogram of acetone extract of Vicia faba peel.

The GC/MS analysis was used to identify the chemical constituents of the n-hexane and acetone fractions of the (VF) peel. The identification was accomplished through the comparison of their spectral fragmentation patterns with those of the available database libraries Wiley and NIST and/or published data in Adams^38^. Table 2 contains an inventory of the identified constituents for the extracts of both hexane and acetone and the results revealed that, the presence of fourteen and thirteen distinct chemical constituents, respectively as mentioned in Figs. 1 and 2. The results of hexane extract proved that 14 compounds were identified in which octacosane and tetratetracontane are the main compounds (27.41 and 19.82% respectively). The other identified compounds are mainly fatty acids and their esters as palmitic acid (11.7%) and ethyl palmitate (12.48%). For the acetone extract, thirteen compounds were identified with heptacosane as a major constituent (42.81%). Also, it was found that some fatty acids are present like lauric acid (5.42%), myrestic acid (5.90%), ethyl palmitate (3.64%), and methyl stearate (2.82%). These data were following reported results by Robert and Wynne^39^.

**Table 2:** GC/MS data of n-hexane and acetone fractions of Vicia faba peel.

Peak no.	R_t_ (min)	%		Mol. Wt	B.P	Molecular formula	Compounds
		Hexane	Acetone				
1	5.16	0.91	–	110	31	C_7_H_10_O	2,4-Heptadienal
2	13.65	1.47	–	184	68	C_12_H_24_O	Z-4-Dodecenol
3	20.07	–	5.42	200	73	C_12_H_24_O_2_	Lauric acid
4	22.31	–	3.16	206	191	C_14_H_22_O	Phenol, 2,4bis(1,1dimethylethyl)
5	25.20	0.87	–	210	43	C_15_H_30_	2,4,6,8Tetramethyl1undecene
6	28.16	–	1.32	228	41	C_15_H_30_O	Pentadecanol
7	28.19	–	5.90	228	73	C_14_H_28_O_2_	Myrestic acid
8	29.30	2.47	–	236	81	C_17_H_32_	1-Heptadecyne
9	30.87	0.72	3.62	240	43	C_17_H_36_	Heptadecane
10	31.78	–	1.22	242	55	C_16_H_34_O	1-Hexadecanol
11	32.09	11.7	–	256	73	C_16_H_32_O_2_	Palmitic acid
12	32.08	–	2.55	270	83	C_18_H_38_O	1-Octadecanol
13	32.20	1.60	–	284	73	C_18_H_36_O_2_	Stearic acid
14	34.56	12.48	3.64	284	88	C_18_H_36_O_2_	Palmitic acid ethyl ester
15	35.07	–	1.77	292	223	C_19_H_16_O_3_	2-Methyl-7hydroxy-8-α llyl-isoflavone
16	35.21	–	3.19	296	43	C17H_36_O	1-Heptadecanol
17	35.51	0.82	2.82	298	74	C_19_H_38_O_2_	Stearic acid methyl ester
18	35.63	0.91	–	312	88	C_20_H_40_O_2_	Stearic acid, ethyl ester
19	35.75	1.87	–	324	57	C_23_H_48_	Tricosane
20	39.03	–	3.25	354	41	C_15_H_30_O_4_	Oxalic acid, methyl dodadecyl ester
21	40.56	1.56	–				
22	41.47	–	42.81	380	57	C_27_H_56_O_4_	Heptacosane
23	43.53	27.41	–	394	57	C_28_H_58_	Octacosane
24	46.27	19.82	–	618	57	C_44_H_90_	Tetratetracontane
Total, %		84.61	80.67				
		14	13

### Electrochemical studies

#### Studies of open circuit potential

**Figure 3:**
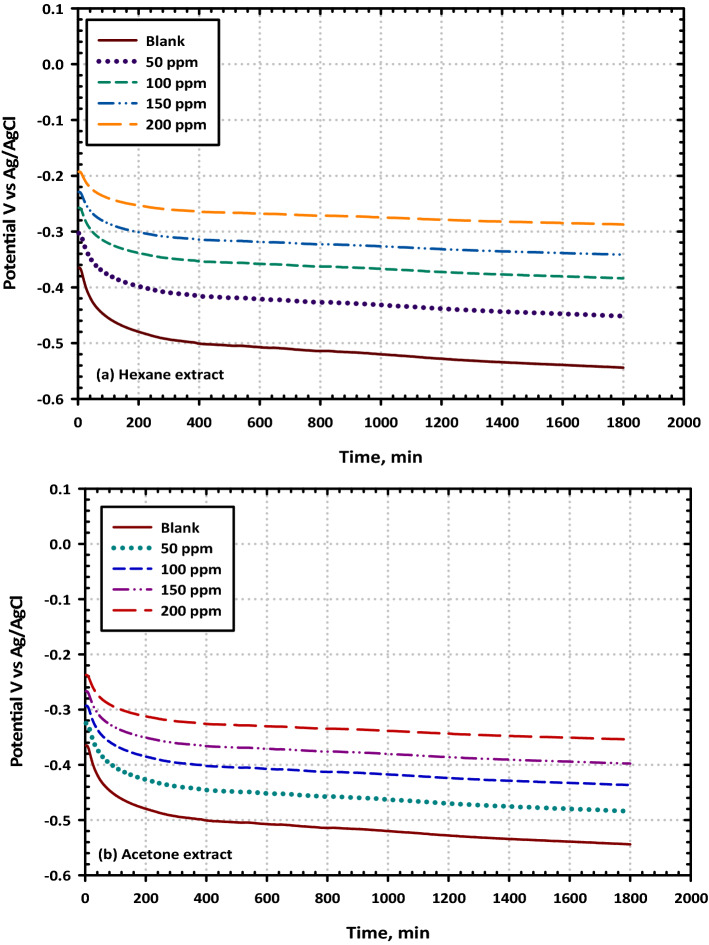
Representative the plots of open circuit potential versus time for mild steel in 3.5% NaCl solution without and with different concentrations (50, 100, 150, and 200 ppm) of (VF) (**a**) Hexane extract and (**b**) Acetone extract at 298 K.

Figure 3A,B illustrate, respectively, the variations in the OCP of the mild steel as a function of time when it was immersed in a 3.5% NaCl solution in the absence and presence of varying amounts of hexane extract Fig. 3a, and acetone extract Fig. 3b, respectively. The equilibrium potential was easily reached, which is equivalent to the free corrosion potential, also known as E_corr_, of the mild steel. The positive charge of the protonated inhibitor and the negative charge of the steel surface both contribute to the process of physical adsorption that takes place in an inhibited solution of 3.5% NaCl. When compared to the unfettered solution, the steady-state E_corr_ value tends to drift toward higher positive values. According to Riggs, it is possible to classify a compound as an anodic or cathodic type inhibitor when the OCP displacement is at least 0.085 V concerning that which was measured for the blank corrosive solution^40^. This criterion must be met before the classification can be considered to be feasible. In contrast, the positive shift in E_corr_ is more than 0.085 V when the examined extracts are present in the environment and acting as a corrosion inhibitor. According to the findings of the OCP, this improvement in E_corr_ is judged to be a significant change that warrants a more appropriate categorization. According to these results, the examined extracts have the effect of lowering the anodic dissolution of iron as well as the hydrogen evolution reaction, and they also induce considerable changes in the values of OCP. Because of this, the two extracts might be considered to be inhibitors of the mixed type^41^. This behavior is in great shape, with a decent agreement with the potentiodynamic polarization observations.

#### Studies of potentiodynamic polarization

**Figure 4:**
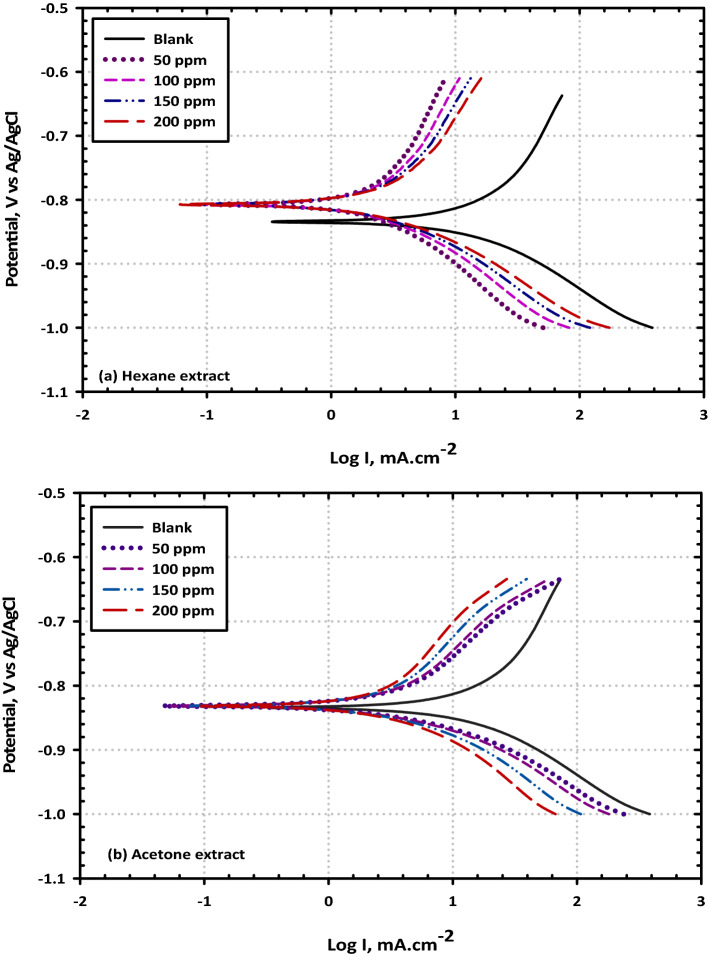
Representative the curves of potentiodynamic polarization for mild steel in 3.5% NaCl solution without and with different concentrations (50, 100, 150, and 200 ppm) of (VF) (**a**) Hexane extract and (**b**) Acetone extract included sweeping the potential between -200 and + 200 mV from OCP at a scan rate of 0.001 V/s at 298 K.

Potentiodynamic polarization is an essential electrochemical instrument for analyzing the many different types of electrochemical corrosion events that may take place on the surfaces of metals. At a temperature of 298 Kelvin, the potentiodynamic polarization measurements were carried out on mild steel in a solution containing 3.5% NaCl as the corrosive medium, both without and with varying concentrations of VF. The corrosion potential (E_corr_), corrosion current densities (i_corr_), anodic and cathodic Tafel slopes (β_a_ and β_c_) for mild steel in 3.5% NaCl medium with and without hexane and/or acetone extracts are mentioned in Fig. 4a,b and reported in Table 3. The addition of extract to 3.5% NaCl solution retards both cathodic and anodic current densities in comparison to the blank saline solution, indicating a mixed-type inhibitive performance. Because the difference in the E_corr_ in the inhibited solution is much lower than 85 mV, the two extracts are classified as mixed-type inhibitors with variance between each other. Potentiodynamic polarization and impedance techniques both have a small degree of disagreement with one another, which is to be expected given that a difference of (± 5) % is typically observed to exist between the results of various tests because experimentation involves a significant amount of variation^42^. Indeed, the values of corrosion potential (E_corr_, mV), corrosion current density (I_corr_, in mA/cm^2^), anodic Tafel slope (β_a_, mV), and cathodic Tafel slope (β_c_, mV) have been deduced by extrapolating from the fitting of the linear Tafel segments of the polarisation curves see Table 3. The polarization resistance (R_p_) could be calculated using the slope of the current density against the potential plot see Eq. (1). According to the findings, it has been concluded that the corrosion potential for mild steel in the absence and the presence of VF extract, is clearly shown that, upon increasing the inhibitor concentration, the corrosion current densities (I_corr_) are proportionally decreased, implying that VF acts as an efficient inhibitor for mild steel corrosion under these conditions with a maximum inhibition efficiency of 97.84% and 88.67% at an inhibitor concentration of 200 ppm of hexane and acetone extracts, respectively. The results of this study have been presented in Table 2. The values of β_a_ and bc shifted as a result of the progressive addition of the inhibitor, which caused the molecules of the inhibitor to adsorb on the surface of the metal and create a thin film of a protective layer. This caused the values of β_a_ and β_c_ to alter and in the presence of a VF inhibitor, it was discovered that the corrosion potential moved in the direction of a smaller negative potential. This is evidence that the adsorption of VF inhibitor across the surface of the metal inhibits both the anodic and the cathodic processes. VF may be categorized as a mixed-type inhibitor with predominant control of the anodic reaction in terms of adsorption mode. This is because the displacement of E_corr_ values was less than 85 mV, and the resultant shift pointed in a more noble direction. The cathodic hydrogen reduction reaction is activation-controlled, and the addition of VF does not alter the mechanism of this process, as shown by the precise analysis of the data that was obtained, which revealed that the cathodic current potential curves give rise to parallel Tafel lines. This finding suggests that the addition of VF does not affect the mechanism of this process but it is noteworthy to notice that the polarisation resistance, or R_p_, increases with an increase in the inhibitor concentration, which suggests that the inhibitor molecules have adsorbed on the active sites of the mild steel surface. Further investigation reveals that this phenomenon has been seen during the building of a covering barrier from the chemical elements of VF between the surface of the metal and the hostile electrolyte may be ascribed to the growing R_p_ values since this barrier protects the metal from further corrosion.

**Table 3:** Potentiodynamic polarization data for mild steel in 3.5% NaCl solution without and with different concentrations (50, 100, 150, and 200 ppm) of (VF) hexane and acetone extracts at 298 K.

Inhibitor	Conc. (ppm)	E_corr_ (mV)	β_a_ (1/V)	β_c_ (1/V)	i_corr_ (µA/cm^2^)	η (%)
Blank	–	− 543	12.521	− 8.396	1290	–
Hexane extract	50	− 446	6.841	− 6.529	196.83	65.55
	100	− 377	4.730	− 5.698	132.02	76.31
	150	− 337	4.496	− 4.541	81.26	87.08
	200	− 285	6.910	− 4.695	58.63	97.84
Acetone extract	50	− 483	7.408	− 7.071	231.16	58.87
	100	− 437	5.483	− 6.605	153.03	86.81
	150	− 396	5.284	− 5.335	95.48	78.73
	200	− 354	8.583	− 5.832	72.83	88.67

#### Studies of electrochemical impedance spectroscopy

Electrochemical impedance spectroscopy EIS was performed to validate the corrosion inhibition ability of VF on the mild steel surface. This was done to conduct a more in-depth analysis of the inhibition process. The findings were acquired from the electrochemical impedance spectroscopy data for the mild steel recorded at open-circuit potential after 30 min of immersion in 3.5% NaCl at 298 K with and without various concentrations of VF.

**Figure 5:**
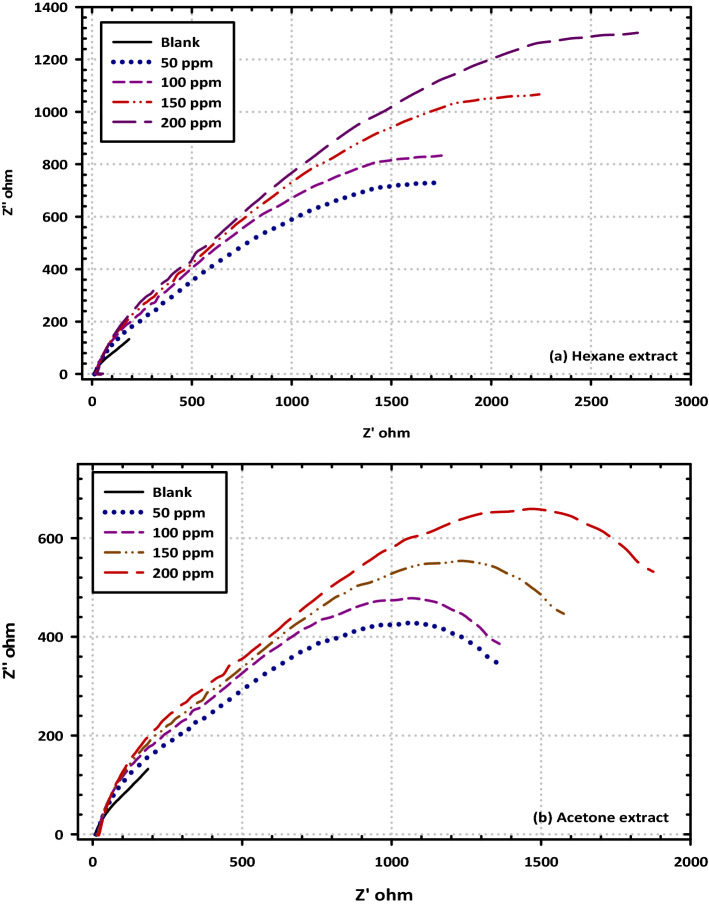
Electrochemical Impedance Spectroscopy (EIS diagrams); Nyquist plots for mild steel in 3.5% NaCl solution without and with different concentrations (50, 100, 150, and 200 ppm) of (**a**) Hexane extract and (**b**) Acetone extract at OCP and 298 K.

**Figure 6:**
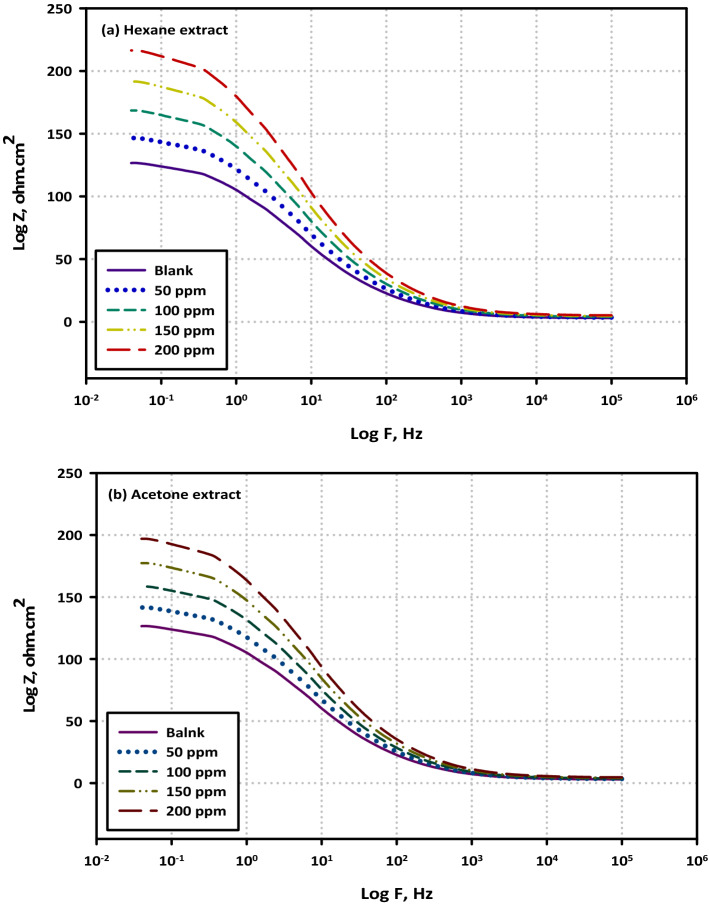
Electrochemical Impedance Spectroscopy (EIS diagrams); Bode plots for mild steel in 3.5% NaCl solution without and with different concentrations (50, 100, 150, and 200 ppm) of (VF) (**a**) Hexane extract and (**b**) Acetone extract at OCP and 298 K.

**Figure 7:**
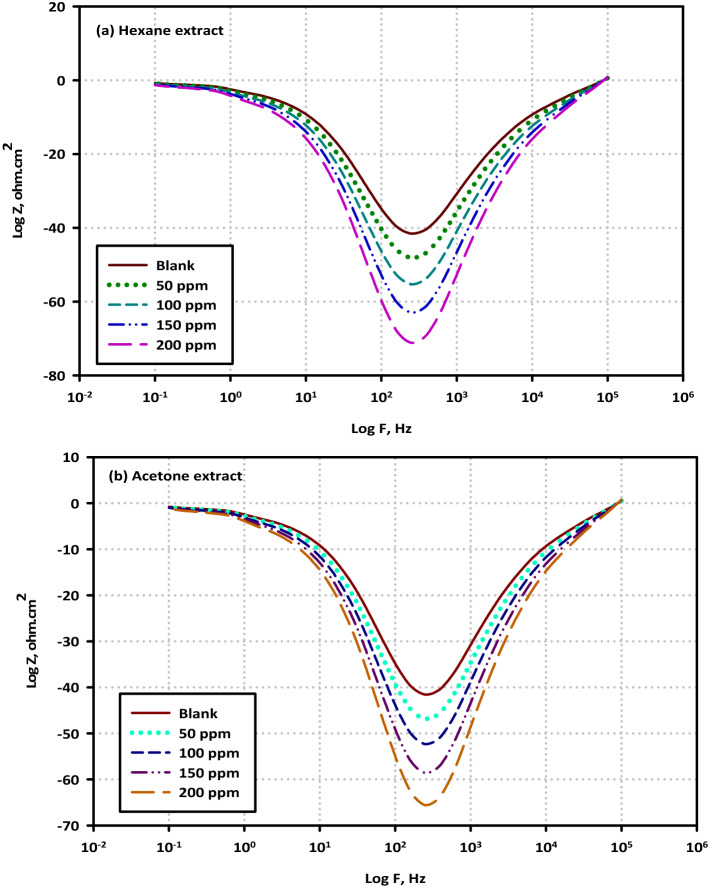
Electrochemical Impedance Spectroscopy (EIS diagrams); Phase plots for mild steel in 3.5% NaCl solution without and with different concentrations (50, 100, 150, and 200 ppm) of (**a**) Hexane extract and (**b**) Acetone extract at OCP and 298 K.

**Figure 8:**
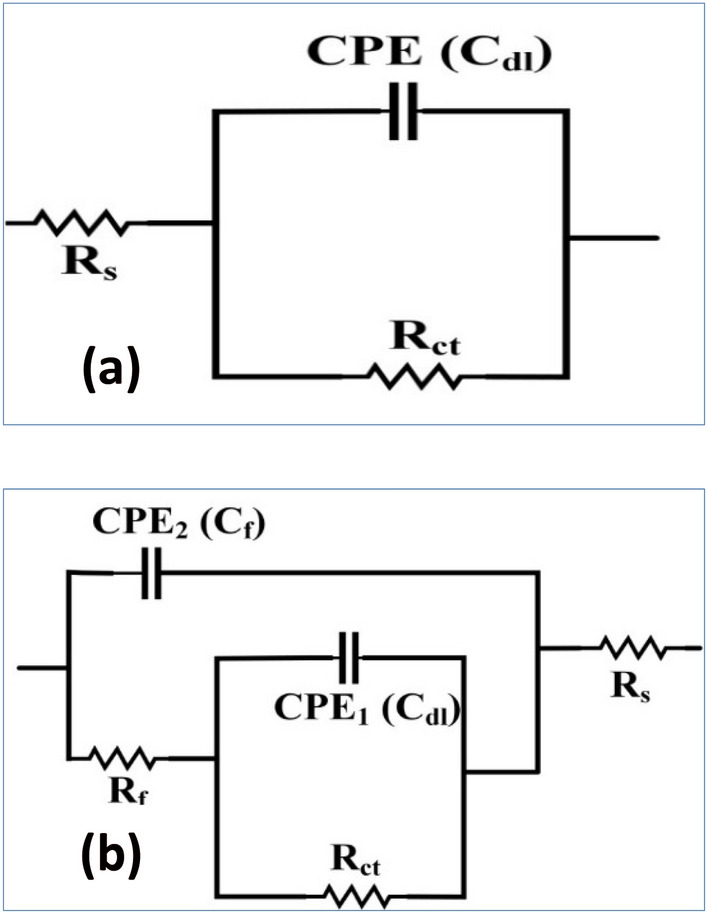
Equivalent circuit of fitting data of mild steel in 3.5% NaCl solution with (VF) (**a**) Hexane extract and (**b**) Acetone extract at OCP and 298 K.

To determine the impedance characteristics of the mild steel/electrolyte interface in the presence of the blank solution as well as various concentrations of each extract, EIS was carried out. The Nyquist plots are mentioned in Fig. 5a,b, The Bode plots are mentioned in Fig. 6a,b, as well as Phase plots, are mentioned in Fig. 7a,b. The equivalent circuits for mild steel without extracts and with extracts are mentioned in Fig. 8a,b. According to the Nyquist plots, the impedance spectra exhibit single depressed semicircles with their centers beneath the real axis. This suggests that the corrosion process was regulated by charger transfer.

These data provide credence to the hypothesis that the electrochemical solid/liquid contact has a non-ideal capacitive tendency in its behavior. In addition, the scale of the impedance diagram becomes larger as the concentration of VF rises, and as a direct consequence of this, the inhibitory effect grows as a result of the adsorption of inhibitor molecules on the surface of the metal It is interesting to note that the depressed semicircles are connected to a distinct departure from ideality at the interface and the consequence of the surface lacking uniformity and roughness, which is a phenomenon that is known as frequency dispersion. The non-ideal capacitive behavior in the experimental data fitting by the electrical equivalent circuit model was depicted by using a constant phase element (CPE). Generally, we were able to produce two capacitive loops, one in the high-frequency region and the other in the low-frequency range, both of which had a single time constant. The creation of a protective barrier layer at the metal/electrolyte interface may be inferred from the Nyquist plot (Fig. 8a,b), which reveals that the charge transfer resistance steadily rises with the addition of a larger concentration of each extract^43^. The amount of charge transfer resistance may be represented by the width of the semicircle in the Nyquist plot. When there is a higher concentration of the extract, there is a corresponding rise in the film's thickness. Due to a certain increase in the thickness of the protective layer, a decrease in the functional area of the surface of the electrode, and a decrease in the dielectric constant, the value of double-layer capacitance (Cdl) decreases when an inhibitor is added to a blank saline solution. Because of this, it may be deduced that the adsorption of VF molecules on the surface of the metal causes a reduction in the surface's heterogeneity. Concerning the double-layer capacitance (Cdl), the findings show that there is a drop in Cdl values following the addition of the VF inhibitor. This can be shown by comparing the results in an inhibitor-free solution to the results in an inhibitor-containing solution. This substantiates the hypothesis that a covering layer was formed on the surface of the electrode. This results in a lower value for Cdl. The value of C_dl_ can be calculated from the following Eq. (2).

2 $${\mathrm{C}}_{\mathrm{dl}}= \frac{{\upvarepsilon }^{0}\mathrm{\varepsilon S}}{\mathrm{d}}$$

where (ε^0^) is the permittivity of air, (ε) is defined as the dielectric constant for the solution, (S) is the functional surface area of the electrode, and (d) is the film's thickness. Since the reduction in the double-layer capacitance may be attributed to either a drop in the dielectric constant (e) or an increase in the interfacial layer thickness, particular focus is placed on the findings (d). Because of the adsorption of the inhibitor molecules on the surface of the mild steel, the reactive surface area has been reduced, which means that the corrosion process has been slowed down. This is the hypothesis that is the most probable to account for these findings. The Bode plot as well as the phase angle plot for mild steel in both the VF-free and VF-containing 3.5% NaCl solutions. Only one time constant is shown in the Bode plots, and that time constant's highest point is located at the intermediate frequency. This is evidence that the charge transfer procedures involve no other processes but relaxing. As the value of the variable of interest (VF) grows, the size of the Bode plots also does. These findings are brought about by the adsorption capacitance of the VF molecules, which rises as the concentration of VF in the solution increases. Because of the frequency dispersion of interfacial impedance, the slopes of Bode plots at intermediate frequencies (S) are not equal to − 1 at any point in the frequency spectrum. In the presence of the VF molecules, the slopes (S) values exhibit a behavior that is capacitive at intermediate frequencies, as shown by the fact that they move toward the negative sign. an increase in the phase angle values seen in the presence of VF molecules as compared to those observed in the blank solution is referred to as an extra explanation. As the volume fraction (VF) concentration grew, so did the phase angle values.

Because of the high adsorption capacity of VF molecules on the surface of mild steel, we may get the conclusion that the presence of VF molecules causes the surface of the mild steel to become smoother. This is because of the interaction between the VF molecules and the surface of the mild steel. The impedance behavior has been described via the use of a straightforward equivalent circuit when there is no inhibitor present, as illustrated in Fig. 8a,b, and another model of a straightforward equivalent circuit when each extract is present. Table 4 contains a listing of the electrochemical characteristics that may be found in the fitted equivalent circuit. Some of these parameters are R_s_ (solution resistance), R_ct_ (charge transfer resistance), and C_dl_ (double-layer capacitance). The following Eq. (3) will be used to determine the effectiveness of inhibition^44^.

**Table 4:** Impedance data for mild steel in 3.5% NaCl solution without and with different concentrations (50, 100, 150, and 200 ppm) of hexane and acetone extracts at 298 K.

Inhibitor	Conc. (ppm)	R_S_ (Ωcm^2^)	R_ct_ (Ωcm^2^)	n	10^6^ Y_0_ (F/cm^2^ s^n−1^)	Fitting error	C_dl_ (µF/cm^2^)	η (%)
Blank	–	4.32	145.46	0.81	415.91	0.07	183.08	–
Hexane extract	50	28.59	1710.36	0.95	27.84	0.17	25.24	74.41
	100	15.27	1742.18	0.96	653.29	0.07	62.64	82.24
	150	9.21	2244.21	0.53	311.19	0.06	90.75	90.07
	200	5.63	2714.52	0.74	13.77	0.12	13.59	97.91
Acetone extract	50	17.69	1058.23	0.97	24.36	0.17	22.24	62.50
	100	9.54	1088.85	0.98	571.62	0.08	54.47	70.21
	150	5.08	1233.37	0.56	272.29	0.07	79.24	77.91
	200	3.07	1480.94	0.74	12.04	0.12	12.20	85.62

3 $$\upeta \left(\mathrm{\%}\right)= \frac{{\mathrm{R}}_{\mathrm{ct}}-{\mathrm{R}}_{\mathrm{ct}}^{0}}{{\mathrm{R}}_{\mathrm{ct}}}\times 100$$

Charge transfer resistance is denoted here by R_ct_ and R^0^_ct_, respectively, depending on whether or not the selected extract is present. The following Eq. (4) is a possible relationship between the components of constant phase element (CPE) and C_dl_

4 $${\mathrm{C}}_{\mathrm{dl}}= {\mathrm{Y}}_{0}^{1/\mathrm{n}}{\mathrm{R}}_{\mathrm{ct}}^{\left(1-\mathrm{n}\right)/\mathrm{n}}$$

where (Y_0_) is the CPE constant and (n) represents the extent of variation from the ideal behavior.

### Microstructure and surface characterization

**Figure 9:**
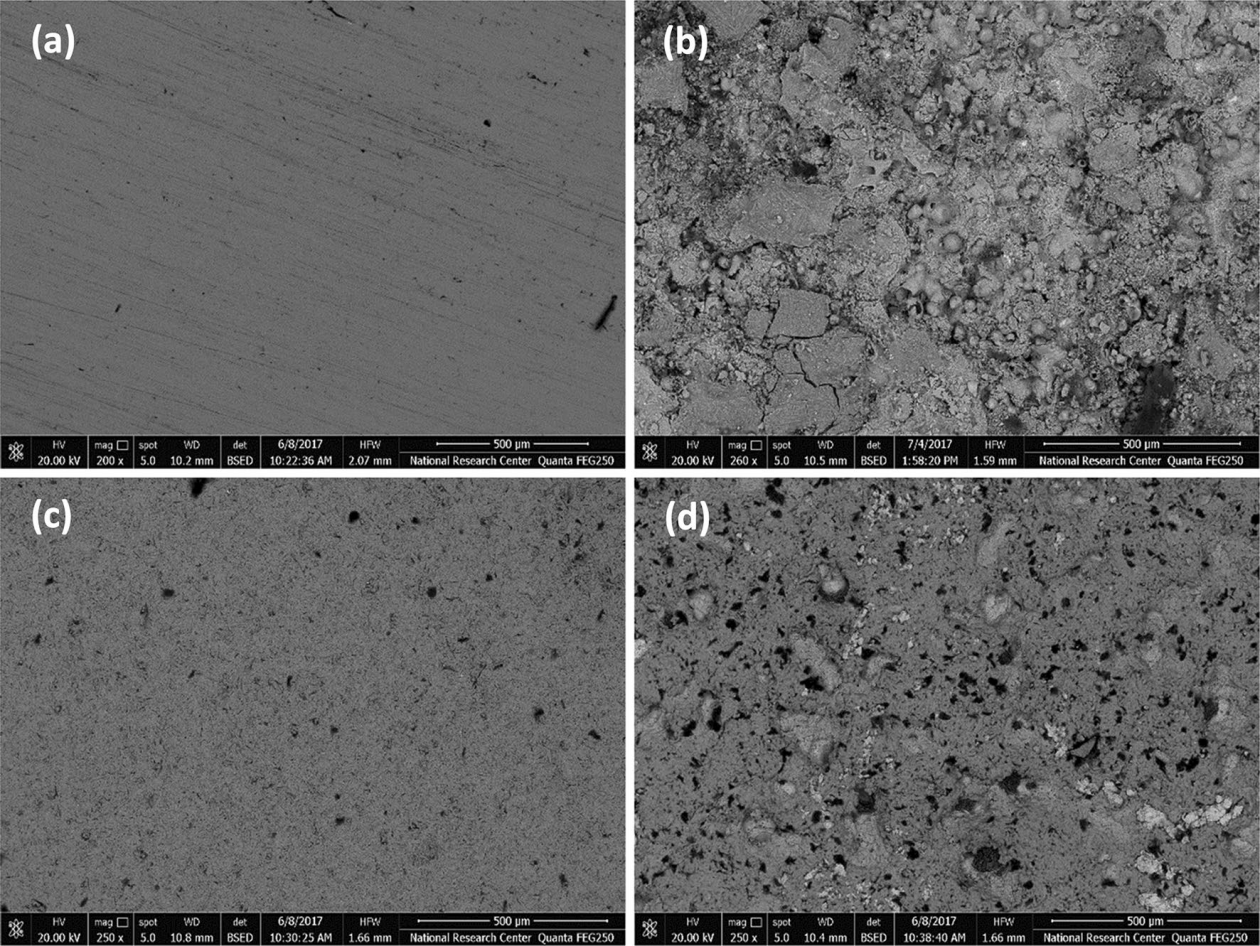
SEM micrograph of mild steel surfaces before and after immersion for 24 h in 3.5% NaCl at 298 ± 1 K, (**a**) Polished mild steel sample, (**b**) In absence of inhibitor (blank), (**c**) in presence of 200 ppm from (VF) hexane extract (**d**) in presence of 200 ppm from (VF) acetone extract.

Figure 9a–d show images taken by a scanning electron microscope of the surface of mild steel after it has been subjected to a medium containing 3.5% NaCl in both the absence and presence of the various extracts. When the polished metal surface (Fig. 9a) was subjected to the blank saline solutions for 24 h without the presence of each extract (Fig. 9b), severe damage was discovered. However, after the addition of 200 ppm of hexane extract at an optimum concentration (Fig. 9c), the smoothness of the metal's surface significantly improved, indicating a reduction in the corrosive attack but in the case of 200 ppm acetone extract (Fig. 9d), it is notable that, the surface is moderately protected compared with the hexane extract. The effect that the roughness of the metal sample has on the adsorption process of metal inhibitors is something that may be readily seen via the use of SEM characterization^45^.

### Effect of temperature on the inhibition efficiency

**Figure 10:**
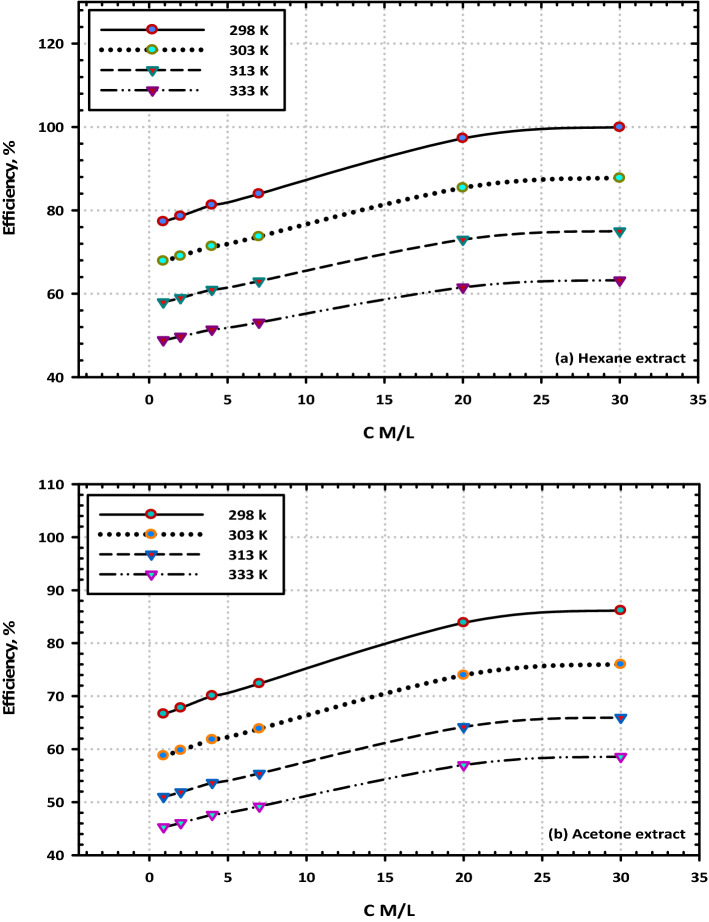
The effect of temperature on the inhibition efficiency of mild steel in 3.5% NaCl at the highest concentration (200 ppm) of (VF) extracts (**a**) Hexane extract and (**b**) Acetone extract.

**Figure 11:**
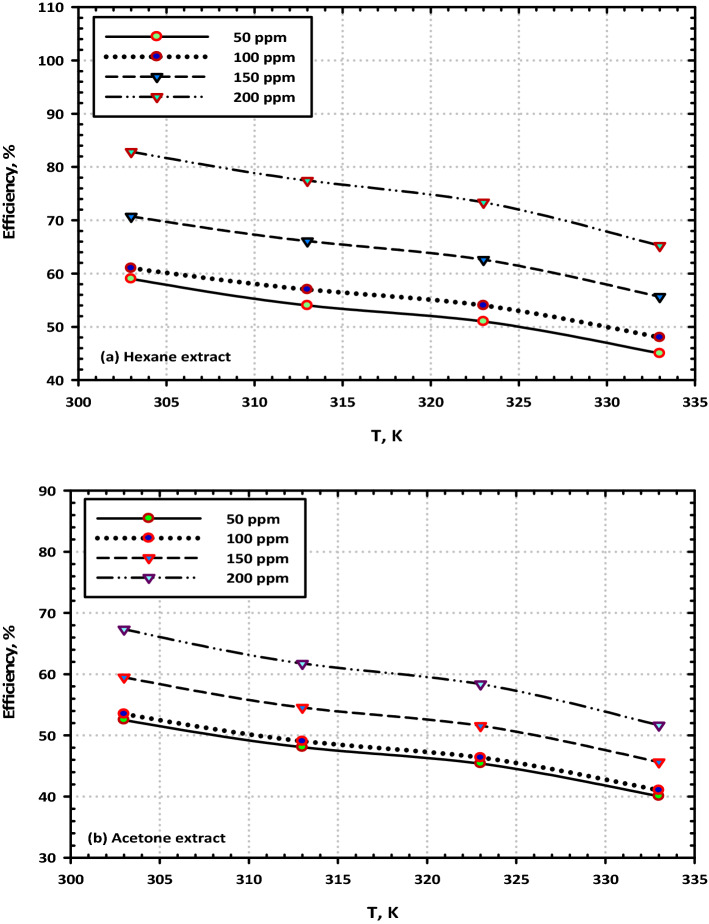
The effect of concentration on the inhibition efficiency of mild steel in 3.5% NaCl at the highest concentration (200 ppm) of (VF) extracts (**a**) Hexane extract and (**b**) Acetone extract.

We investigated how the concentration of the (VF) extracts as well as the temperature influenced the effectiveness of the corrosion-inhibitory properties of the extracts. A variation in the inhibitory efficiency as a function of the concentration of hexane and acetone extracts is shown in Fig. 10a,b and can be seen to occur over a range of temperatures. As can be observed, the inhibitory effectiveness grows with increasing concentrations of both extracts, and this holds across all temperatures. The relationship of the inhibitory efficiency to the temperature of the electrolyte is shown in Fig. 11a,b for a variety of concentrations of (VF) extracts. It has been shown that the temperature harms the corrosion inhibition effectiveness of (VF) extracts, even when the content of the extracts remains the same. When the extracts of hexane and acetone are at greater concentrations, namely 200 ppm, the magnitude of the detrimental effect that temperature has on the effectiveness of the inhibition is reduced^46^.

### Adsorption isotherms

Adsorption isotherm investigations were carried out so that the nature of the interaction between the species present in the (VF) extracts and the surface of the mild steel could be investigated. There are several adsorption isotherms, such as the Langmuir, Freundlich, and Temkin isotherms that may be used to characterize the connection between the amount of surface coverage and the number of species that have been adsorbed. It was discovered that the adsorptive interaction between two extracts followed the Langmuir isotherm^47^. The Eq. (5) that may be used to define the Langmuir adsorption isotherm is as follows:

5 $$\frac{{C}_{inb}}{\theta }=\frac{1}{{k}_{ads}}+{C}_{inb}$$

where (θ) is the surface covering degree, (C_inb_) is the concentration of the utilized extract, and (K_ads_) is the adsorption equilibrium constant that was derived earlier through PD and EIS measurements.

**Figure 12:**
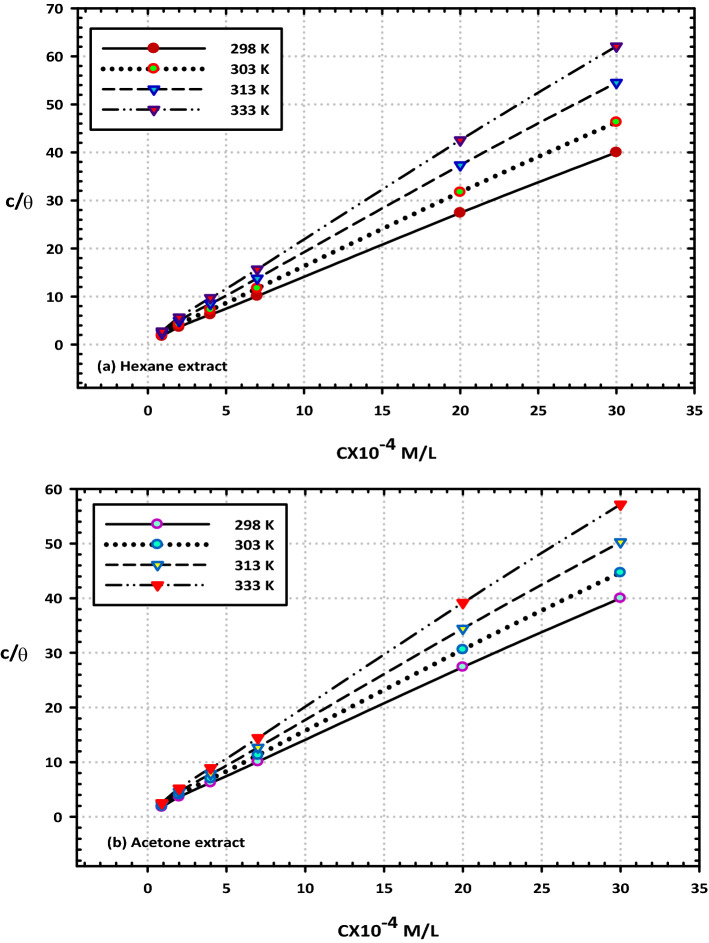
Representative the Langmuir isotherms for the adsorption of mild steel in 3.5% sodium chloride in the presence of (200 ppm) from (**a**) hexane and (**b**) acetone extracts.

When the Langmuir connection between C_inb_/ and C_inb_ is plotted, the result is a significant straight line with a correlation coefficient of one and an average slope value of 1.35, as shown in Fig. 12a,b. Therefore, there is proof that the species that make up the extract ingredients are adsorbed without any side interactions occurring. Using the intercept of the C_inb_/ against C_inb_ line in Table 5, we were able to compute the equilibrium constant for extract adsorption, which is denoted by the symbol K_ads_. As a consequence of this, the value of K_ads_ was used to calculate the adsorption standard free energy (${G}_{ads}^{o}$) using the following Eq. (6):

**Table 5:** Adsorption isotherms parameters of hexane and acetone extracts.

Extract	T (K)	R^2^	slope	*K*_*ads*_, (M^−1^)	∆${G}_{ads}^{o}$ (KJ/mol)	∆${H}_{ads}^{o}$ (KJ/mol)	∆${S}_{ads}^{o}$ (J/mole/K)
Hexane extract	298 303 313 333	0.9998 0.9998 0.9998 0.9998	0.954 1.277 1.773 2.299	11,059 3147 2018 1351	− 33.8 − 31.6 − 31.6 − 31.6	− 71.98	− 159.6
Acetone extract	298 303 313 333	0.9998 0.9998 0.9998 0.9996	0.954 1.190 1.507 1.948	11,059 3141 2004 1342	− 32.7 − 29.6 − 29.6 − 29.6	− 69.52	− 146.2

6 $${ka}_{ads}=\frac{1}{55.5}\mathrm{exp}(-\frac{{\Delta G}_{ads}^{o}}{RT})$$

According to what is shown in Table 5, the values of ${G}_{ads}^{o}$ were calculated for the two extracts when they were used as a corrosion inhibitors at various temperatures. It can be shown that the high negative values of ${G}_{ads}^{o}$ are associated with the impulsive adsorption performance of extracts on the surface, and these values pertain to the steady state of the adsorbed layer. This phenomenon is indicative of the intense interaction that is taking place between the contents of the two extracts and the electrode surface^48^. The values of ${G}_{ads}^{o}$ up to 20 kJ mol^−1^ are linked with the electrostatic interaction of the charged compounds with the charged surface. The adsorption process, in this instance, is just a physical one. This is a common idea. The presence of chemical adsorption will be seen if the values of ${G}_{ads}^{o}$ are increased to be more than − 40 kJ/mol. As a result of the measurements that were taken, the values of ${G}_{ads}^{o}$ were determined to be stable at a value of − 31 kJ/mol in the temperature range of 298–333 K. This provides evidence that the adsorption of species by extracts of hexane and acetone are typical examples of physical adsorption. According to the findings, the interaction between the active cation of the extract ingredients and the charged centers on the electrode surface is what causes the adsorption of the species present in the two extracts, which is typically the cation. This interaction takes place physically and electrostatically^49^. The fact that the values of ${G}_{ads}^{o}$ are negative is a strong indicator of the spontaneous character of the adsorption of the extract species on the surface. Additionally, the heat of adsorption, denoted by the symbol ${H}_{ads}^{o}$, was calculated using the Van't Hoff Eq. (7):

7 $$\ln {\text{K}}_{{{\text{ads}}}} = - \frac{{\Delta {\text{H}}_{{{\text{ads}}}}^{{\text{o}}} }}{{{\text{RT}}}} + {\text{A}}$$

**Figure 13:**
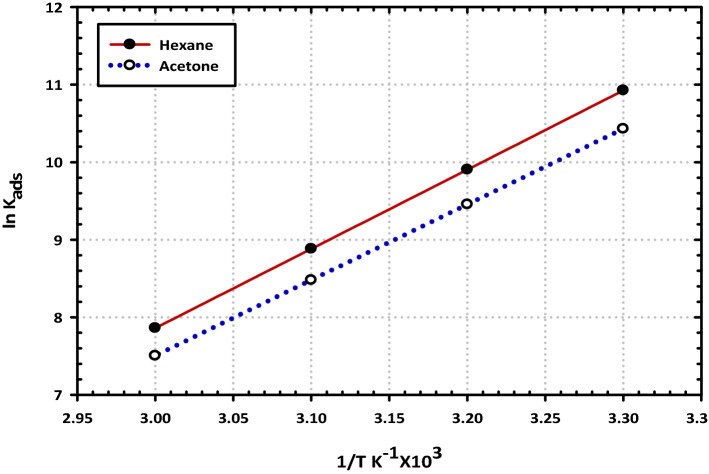
Representative the Temkin isotherms for the adsorption of mild steel in 3.5% sodium chloride solution in the presence of (200 ppm) from the (**a**) hexane and (**b**) acetone extracts.

When the relationship between ln K_ads_ and 1/T is plotted against one another, a straight line is formed, as can be seen, shown in Fig. 13. The slope of the resulting line has a value that is comparable to (${H}_{ads}^{o}$/R), and the adsorption heat value (${H}_{ads}^{o}$) is very close to the standard adsorption heat value following the methods for testing. The equation that must be used to calculate the standard entropy of adsorption, which is denoted by the symbol, ${S}_{ads}^{o}$, can be found in thermodynamics and reads as follows:

8 $$\Delta {\text{S}}_{{{\text{ads}}}}^{{\text{o}}} = \frac{{\Delta {\text{H}}_{{{\text{ads}}}}^{{\text{o}}} - \Delta {\text{G}}_{{{\text{ads}}}}^{{\text{o}}} }}{{\text{T}}}$$

Table 5 contains a compilation of the thermodynamic parameters that were determined, including, ${G}_{ads}^{o}$, ${H}_{ads}^{o}$, and ${S}_{ads}^{o}$. It can be observed that the '${H}_{ads}^{o}$ value is negative (− 73 KJ/mole), which indicates that there is an effect caused by the adsorption of extracts on the surface. When one examines the values of (${S}_{ads}^{o}$) that are shown in Table 5, it becomes immediately apparent that the value of (${S}_{ads}^{o}$) has a negative sign (− 159.6 and − 146.2 J/mole/K)^50^. The negative signal of ${S}_{ads}^{o}$ may be associated with a medium that is dissolute, which is often shown to be an increase in disorder since the reactants are changing into efficient complexes. Moreover, the observed way may be explained by the replacement mechanism of more water molecules during the process of extracts being adsorbed onto the surface. This takes place throughout the adsorption process^51^.

### Kinetic activation

In addition to the research into thermodynamics, a kinetic activation model is also an important tool that can be used to examine the mechanism of action of corrosion inhibition and explain the characteristics of the protective effect that (VF) extracts have at various temperatures^52^. To estimate the activation parameters of the corrosion, the Arrhenius equation was used, see Eq. (9):

9 $$\ln {\text{CR}} = \frac{{{\text{E}}_{{\text{a}}} }}{{{\text{RT}}}} \times \ln {\text{A}}$$

where (CR) stands for the rate of corrosion, (E_a_) for the energy needed to activate, (R) for the constant of the gas, and (A) for the pre-exponential factor.

**Figure 14:**
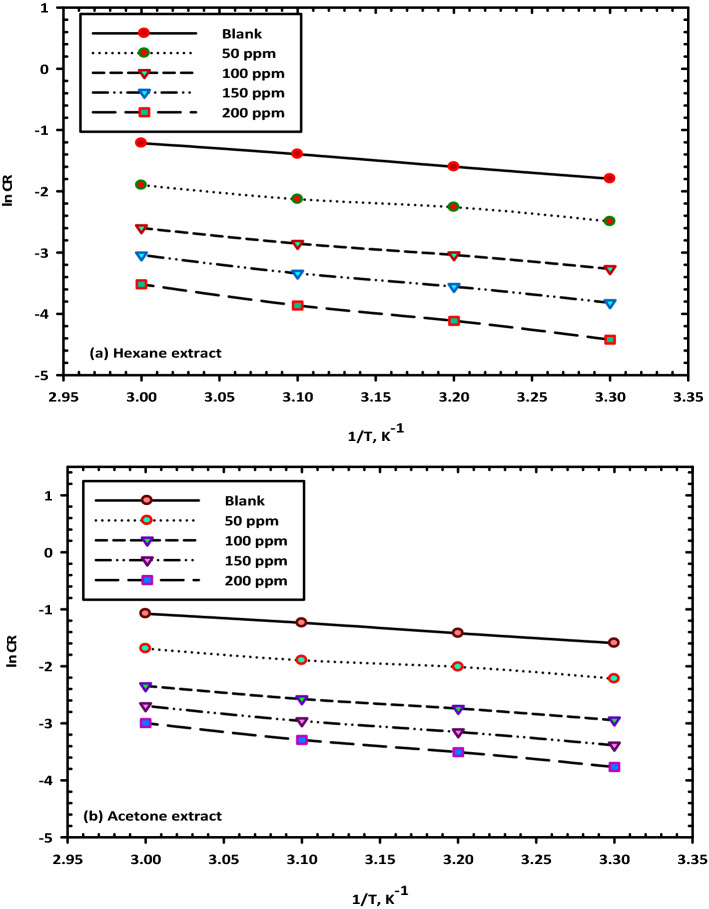
Arrhenius plots for the corrosion of mild steel in 3.5% NaCl solution with several concentrations of (VF) (**a**) hexane and (**b**) acetone extract.

Figure 14a,b illustrate the Arrhenius plots illustrating the link between the logarithm of the temperature, or 1/T, and the CR. As can be observed, a linear behavior is produced, and the slopes of the straight lines are (− E_a_/R); thus, the values of the activation energy (E_a_) were measured in both the presence and absence of different concentrations of (VF) extracts. In addition, there is still another formulation for the transition state, which is outlined in Eq. (10) which is shown below^53^.

10 $$\ln {\text{CR}} = \frac{{{\text{RT}}}}{{{\text{Nh}}}}\exp \left\{ {\frac{{\Delta {\text{S}}^{*} }}{{\text{R}}}} \right\}\exp \left\{ {\frac{{ - \Delta {\text{H}}^{*} }}{{{\text{RT}}}}} \right\}$$

**Figure 15:**
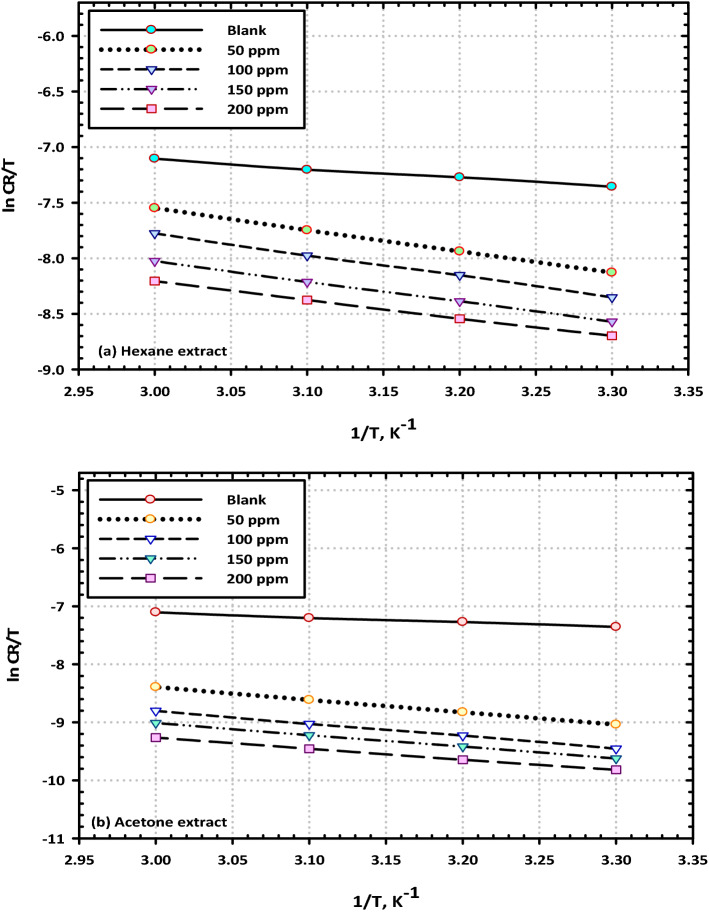
Transition-state plots for mild steel corrosion in 3.5% NaCl solution with several concentrations of (VF) (**a**) hexane and (**b**) acetone extract.

Avogadro's number (N), the activation entropy (S*), and the activation enthalpy (H*) are the terms that make up the equation. Plank's constant (h) is also one of the components. In Fig. 15a,b, the plots of ln CR/T vs 1/T for the (VF) extract as a corrosion inhibitor are shown for the reader's perusal. As a result of the fact that the slope of the produced lines is equal to (− H*/R) and the intercept is (lnR/Nh + S*/R), it is possible to estimate the values of H* and S*. The activation parameters for mild steel in sodium chloride medium have been obtained and displayed in Table 6. These values are determined at certain concentration ranges of the (VF) extracts as a corrosion inhibitor. According to the findings, the presence of (VF) extracts causes an increase in the activation energy while simultaneously causing a modest decrease in the activation enthalpy^54^. At the same time, the values of the entropy for the corrosion process dramatically increase. At a high concentration of (VF) extract contents, the energy of activation (E_a_), which is the minimum amount of energy required to start a chemical reaction, reaches a value of 21 kJ/mol, whereas the value for the blank solution is only 16 kJ/mol. This indicates that the amount of energy required to start a chemical reaction is proportional to the concentration of the reactant. The physical adsorption process may be responsible for the rise in the E_a_ value that occurred as a result of the action of (VF) extracts as a corrosion inhibitor. According to a further explanation, the rising of the activation energy value can potentially be related to the progressive decrease in the adsorption process of (VF) extracts on the surface under the influence of heat^55^. This was cited as a possible reason since based on these events, when the adsorption process slows down, an increase in the desorption action of (VF) extract species takes place as the protection and dissolution systems reach a state of equilibrium. It was also discovered that the successive values of E_a_ and H^o^ as reported in Table 6 are altered in the same pattern, which is confirmation that the thermodynamic processes in question are shared by both systems^56^. The value of the activation entropies has been reported to be negative, which indicates that the activated complex is in the rate-determining stage and displaying combination rather than separation and this was discovered by specific monitoring^57^.

**Table 6:** Activation energy parameters at different concentrations of hexane and acetone extract.

Extract	Conc. (ppm)	E_a_ (KJ/mol)	∆H^o^_a_ (KJ/mol)	∆S^o^_a_ (J/mol/K)
	Blank	16	7	− 236
Hexane extract	50 100 150 200	16 19 20 21	16 16 15 14	− 212 − 215 − 219 − 225
Acetone extract	50 100 150 200	15 16 19 20	15 16 16 15	− 213 − 212 − 215 − 219

### Theoretical assay

**Figure 16:**
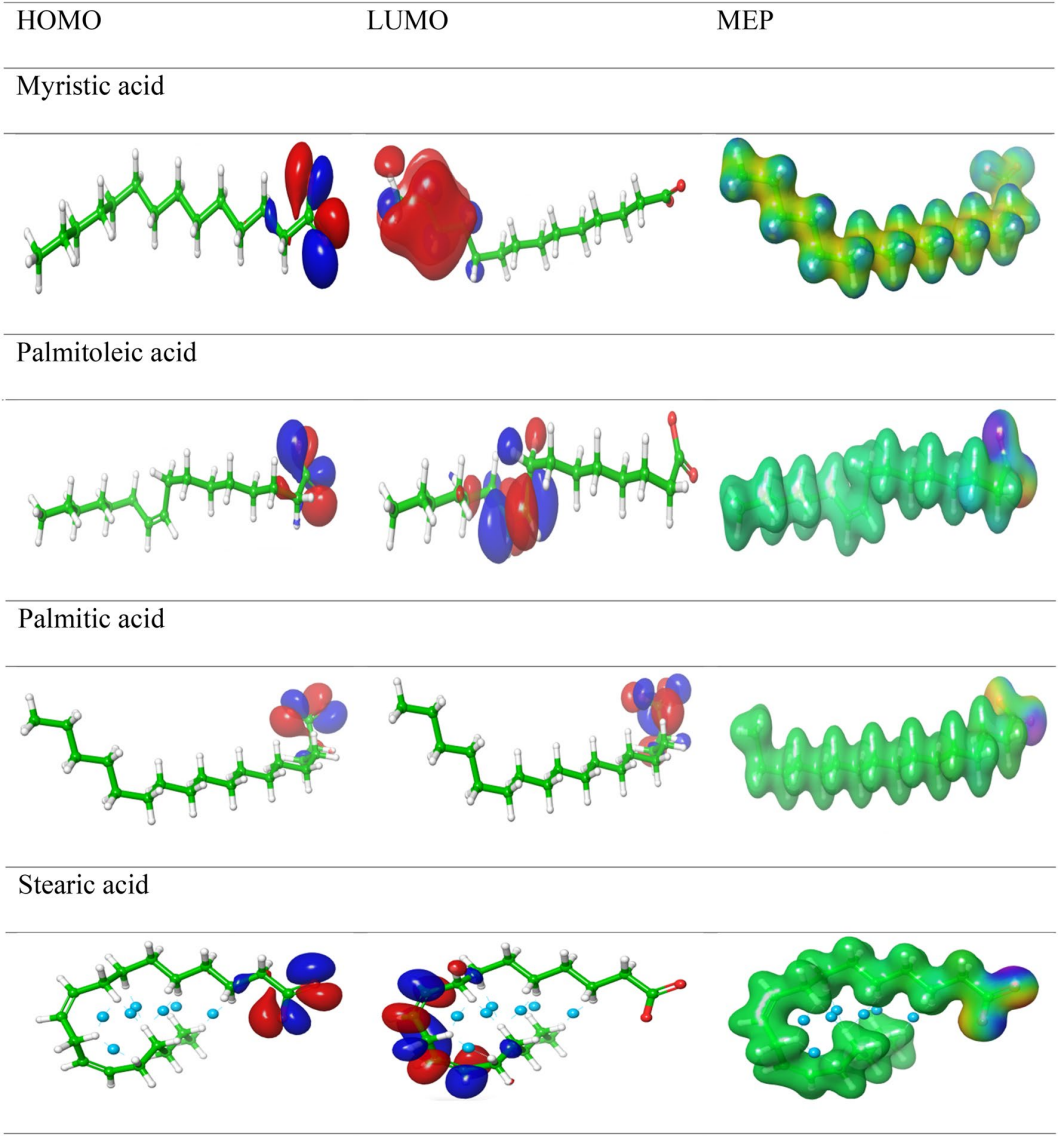
Structure optimization and FMOs obtained by distributions of (HOMO) and (LUMO) at the major constituents of (VF) obtained by DFT/B3YLB/6-311G** for AO and HERA.

DFT theory using B3LYP/6-311G** was used for the computational study of the five fatty acids extracted from (VF). Figure 16 showed the optimized HOMO and LUMO and MEPs for the tested compounds. The chemical parameters were calculated using equations as reported and listed in Table 7^58^.

**Table 7:** Calculated reactivity parameters for Fatty acid at DFT/B3YLB/6-311G**.

	HOMO (au.)	LUMO (au.)	ΔG (au.)	DM (D)	η (au.)	S (au.)	χ (au.)	I (au.)	A (au.)	EP (au.)	ωi (au.)	µ+ (au.)	µ− (au.)	ω− (au.)	ω+ (au.)	ΔEBD (au.)	ΔNmax (au.)
**(Gas phase)**																	
Myristic	− 0.07	− 0.13	0.06	36.37	0.06	16.12	0.06	− 0.01	− 0.13	− 0.07	0.03	0.10	0.04	0.01	0.03	− 0.02	− 0.55
Palmitoleic	− 0.06	− 0.16	− 0.1	32.07	0.04	25.19	0.04	− 0.01	− 0.09	− 0.05	0.02	0.07	0.03	0.01	0.02	− 0.01	− 0.58
Palmitic	− 0.08	− 0.12	− 0.04	35.13	0.06	16.51	0.06	− 0.01	− 0.13	− 0.07	0.03	0.10	0.04	0.01	0.03	− 0.02	− 0.57
Stearic	− 0.09	− 0.13	− 0.04	33.21	0.06	15.71	0.06	− 0.01	− 0.14	− 0.07	0.03	0.10	0.04	0.01	0.03	− 0.02	− 0.57
Myristic	− 0.061	− 0.18	− 0.119	24.58	0.03	28.58	0.03	− 0.01	− 0.08	− 0.04	0.02	0.06	0.02	0.01	0.02	− 0.01	− 0.59
**(Aqueous phase)**																	
Myristic	− 0.23	− 0.07	0.16	39.16	0.15	6.68	0.15	0.23	− 0.07	0.08	0.00	− 0.01	− 0.15	0.08	0.00	− 0.04	− 0.28
Palmitoleic	− 0.25	− 0.02	0.23	35.47	0.13	7.99	0.13	0.23	− 0.02	0.10	0.03	− 0.04	− 0.17	0.11	0.03	− 0.03	− 0.41
Palmitic	− 0.27	− 0.05	0.22	38.39	0.16	6.78	0.15	0.23	− 0.07	0.08	0.00	− 0.01	− 0.15	0.08	0.00	− 0.04	− 0.28
Stearic acid	− 0.28	− 0.01	0.27	28.38	0.12	8.39	0.12	0.23	− 0.01	0.11	0.04	− 0.05	− 0.17	0.12	0.04	− 0.03	− 0.46

#### Stability inter and intermolecular interaction against Fe surface

##### "FMOs" frontier molecular orbitals analysis

HOMO and LUMO are known as "FMOs" and are defined by donating/accepting electrons, respectively, which may determine the path binding of fatty acid with a steel surface^59–61^. The FMO gap is used to measure a molecule's chemical reactivity and kinetic stability. Raising the inhibitor's HOMO energy and reducing the surface's LUMO energy improved (inhibitor-surface) stabilization^62–64^. E_HOMO_ in the aqueous phase is higher than in the gas phase and the component is arranged in decreasing order as follows Palmitic acid > Palmitoleic > Myristic acid > Stearic (Table 7). The greater E_HOMO_ value than E_LUMO_ indicates a greater likelihood of losing valance electrons and a greater tendency to donate electrons toward the surface of iron, and more inhibition potency than the gas phase^65–67^. The HOMOs were localized over all carboxy groups in all tested fatty acids while LUMOs tagged over the aliphatic group (Fig. 16). The negative E_HOMOs_ and E_LUMOs_ indicated that the chargeable migrated from Fatty acid to the Fe surface, which was penetrated by the carboxylic centers.

##### “ESP” molecular electrostatic potential profile

In the adsorption process; ESP for the fatty acid inhibitors can identify of Fe adsorption site, by finding the balance between scattering and appealing interactions^68–71^. The scattering force (nuclei with “+” charge) signifies a blue color and caused electron donation power. The attractive force with (−) charge, is associated with electron-accepting and expressed as (orange, yellow, and red). The green color point to an intermediate potential value. ESP has been graphed for Fatty acid in (Fig. 16). The negative charges were allocated as follows: Myristic acid > Palmitic acid > Stearic acid > Palmitoleic acid. ESP surface's color variation showed the variation for their values. The highest negative region is related to high the efficacy of their penetration effect over the Fe surface.

#### Global chemical reactivity

The interaction between HOMO_inhibitor_ and LUMO_surface_ has indirectly stabilized with an energy gap. The stability index for Fatty acid adhering to Fe surfaces was determined by ΔG. Fatty acid exhibited lower ΔG values in the gas phase than liquid phase, respectively. These values showed promising reactivity liquid phase (Table 8). Furthermore, ΔG related linearly with the soft-nucleophile and hard-electrophile. Thus, the molecule which has a little ΔG displayed promising softness properties, which are good inhibitors in acidic media^72–74^. The data obtained from (Table 8), demonstrated greater softness values in the liquid phase than in the gas phase. Also, we examined the number of electrons transferred from the inhibitor (donor) to the Fe (acceptor) by calculating the (μ− & ω−)^75–78^. The inhibition action increases by growing value for (μ− & ω−) which refers to the high ability for donating electrons by the inhibitor, respectively, and vice versa for (μ+ & ω+). The liquid phase exhibited a high capacity of the sharing electron than the gas phase. Besides, the highest electron amount transferred (ΔN_max_) for the liquid phase (0.055–0.059) is more than the gas phase (− 0.28 to − 0.46), which supported the claimed experimental findings of high efficiency for Fatty acid in the liquid phase. The process of the electron contribution as a (Fe → inhibitor) is recognized as energy back donation. (ΔE_BD_), which determined the (Inhibitor-Fe_surface_) interaction. The higher ΔE_BD_ in the liquid phase than in the gas phase displayed a promising penetration ability with higher inhibition efficiency for the liquid phase. The negative values of (ΔE_BD_, μ+, ω+) proposed that the (Fe → inhibitor) electron follows in the liquid phase are more favorable energy than gas phase (Table 8). These findings were consistent with the experimental data^79–82^.

**Table 8:** The calculated descriptors (kcal/mol) of Fatty acid on Fe (100).

Inhibitor energy	Myristic acid (C14:0)	Palmitoleic acid (C16:1)	Palmitic acid (C16:0)	Stearic acid (C18:0)
Total energy	− 25.02	− 19.70	− 25.02	− 33.12
Adsorption energy	− 3.50	− 4.178	− 4.50	− 4.99
Rigid adsorption energy	− 37.70	− 35.64	− 71.01	− 49.68
d_Ead_/d_Ni_	− 4.50	− 5.17	− 6.17	− 5.99

#### Molecular dynamic (MD) profile

**Figure 17:**
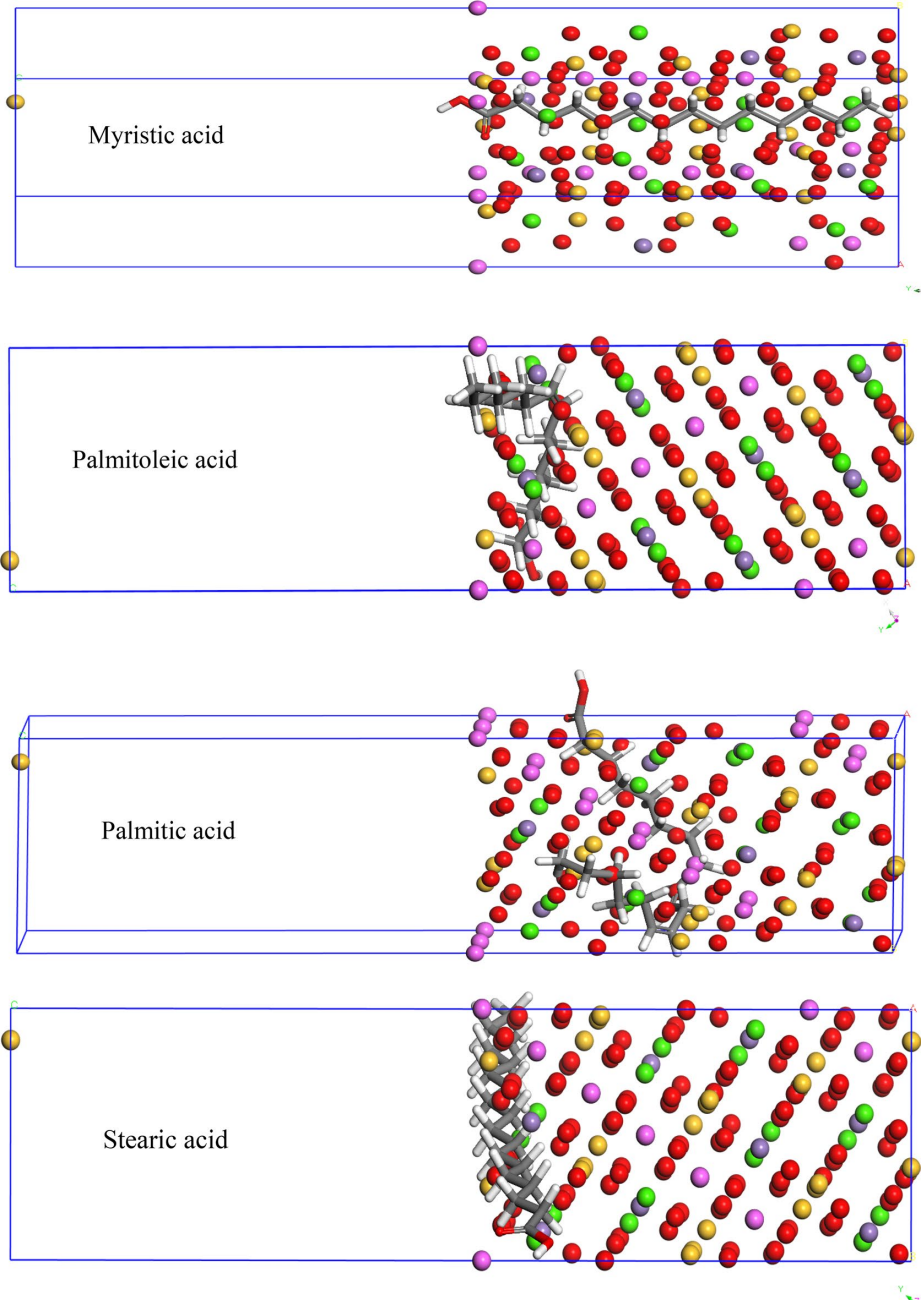
Representative the side view of molecular simulations for the most favorable modes of adsorption mode for the inhibitors on the Fe (100) surface.

(MD) the simulation was performed to provide a better understanding of the (Inhibitor-Fe) interaction (Fig. 17). (Adsorption Locator Model) was utilized to recognize the ideal adsorption site for mild steel surface against Fatty acid inhibitors. Thus; the lowest inhibition energy was listed in (Table 8); as follows; total energy for the substrate, rigid adsorption energy (unrelaxed tested components which adsorbed on Fe metal), adsorption energy(rigid adsorption & deformation energies), (dE_ads_/d_Ni_) (Fe and Fatty acid inhibitors energy; where one of the inhibitors was removed), binding energy (the negative value of adsorption energy)^83–85^.

In the stable configuration (Fig. 17), Palmitoleic, and stearic acids were stabilized in parallel to the mild steel surface (100) plane, while Myristic and Palmitic acids were arranged in the plane to mild steel (100). In addition, the adsorption energy is arranged as Stearic > Palmitic > Palmitoleic > Myristic acids for (Table 8). This theoretical vision is in line with experimental findings. The located electron lone pair over O atoms of Fatty acid inhibitors caused high stability for coordination interaction (inhibitor → Fe). From (Fig. 17) we can observe that Fatty acid inhibitors can adsorb into the mild steel by the carboxyl group of Fatty acid inhibitors. Thus, will make a mild steel surface do not interact with the acid solution^86–88^.

### Mechanism of corrosion inhibition

The presence of a significant ingredient of (VF) extracts obtained from hexane and acetone delayed the corrosion of mild steel in 3.5% sodium chloride. The mechanism of inhibition is catalyzed by the presence of two heterocyclic rings among the main ingredients. Nitrogen and delocalized pi electrons interact with the surface of the metal to generate a strong and shielding adsorption layer over the metal^89–92^. The presence of donor atoms, such as nitrogen, is the primary factor that determines the extent to which adsorption is impacted. Iron (Fe) has some empty d orbitals, which are happy to take the electrons that are being provided by the inhibitor. Because of the adsorption of chloride anions that are present in the NaCl solution, the surface of the metal becomes negatively charged. Because of this, it can draw near itself the protonated form of heterocyclic rings and heteroatoms. To create a chemisorbed layer, the neutral molecules that are present in the primary elements of each extract must first displace the molecules of water that are adsorbed to the surface of the metal. Inhibition of corrosion of mild steel by either extract in a solution containing 3.5% sodium chloride may be rationalized based on molecular adsorption^93–95^. These chemicals prevent corrosion by regulating the processes that occur during both the anodic and cathodic phases. According to what was covered in the section on adsorption isotherms, both extracts were chemically adsorbed on the surface of the mild steel. The extracts exist as protonated species in the saline solution that has been inhibited^96–98^. These protonated species are responsible for the reduction in the evolution of hydrogen as they adsorb on the cathodic sites of the mild steel. The adsorption of electrons on the anodic site occurs due to the presence of lone pairs of electrons on oxygen atoms and -electrons on benzene rings and heterocyclic compounds, all of which are found in both extracts. This results in a reduction in the anodic dissolution of mild steel^99–102^.

## Conclusions

Different chemical components of *Vicia faba* peels were extracted using hexane and acetone, and their identities were determined in this research. Anti-corrosion protection for mild steel in 3.5% NaCl was achieved using the two extracts. Various electrochemical techniques, including open circuit potential (OCP), potentiodynamic polarization (PD), and electrochemical impedance spectroscopy (EIS), were used to study the corrosion behavior of mild steel in a salty environment (EIS). Inhibitors of both cathodic and anodic corrosion currents, as shown by the OCP, PD, and EIS curves, the two extracts are classified as mixed-type inhibitors. Based on the electrochemical readings, it was clear that the presence of two extracts improved the efficacy of the inhibition. This was because the electronic density of the inhibitor molecules increased as a result of the presence of benzene rings in the extracts' and molecules' primary components. Adsorption is responsible for the inhibition, and this adsorption has been shown to follow the Langmuir adsorption isotherm. The calculated Gibbs free energy of adsorption further supports a chemical basis for the adsorption. Corrosion of mild steel has been studied in a medium containing 3.5% NaCl, where the inhibitory powers of hexane and acetone extract (VF) have been analyzed. Electrochemical testing and scanning electron microscopy (SEM) analyses have been used in this study. Results from the trials show that 200 ppm of either the hexane or acetone extracts is optimal for maximum efficiency. Physisorption is the mechanism in play, as shown by the fact that G ads 0 equals − 10.71 kJ/mol and the Langmuir adsorption isotherm is respected. When inhibitors are present, Ecorr may move significantly; this variation demonstrates that the studied inhibitors are a composite of several types. If you look at the blank and then at the inclusion of the inhibitor, you'll see what I mean. Moreover, scanning electron micrographs (SEMs) demonstrate the greater activity of the protective layer barrier formed by inhibitors on the surface of the mild steel. The results of the theoretical study provide greater credence to the inhibitory performance seen here.

## Data Availability

All data generated or analyzed during this study are included in this published article.
